# Clinical report and genetic analysis of a Chinese patient with developmental and epileptic encephalopathy associated with novel biallelic variants in the *ST3GAL3* gene

**DOI:** 10.1002/mgg3.2322

**Published:** 2023-11-08

**Authors:** Jihong Hu, Juan Liu, Chunguang Guo, Yaqin Duan, Chunlei Liu, Yaqiong Tan, Ying Pan

**Affiliations:** ^1^ Department of Rehabilitation Hunan Children's Hospital Changsha China

**Keywords:** developmental and epileptic encephalopathy, neurodevelopmental disorders, ST3GAL3, ST3Gal‐III

## Abstract

**Background:**

Defects in the Golgi enzyme beta‐galactoside‐alpha‐2,3‐sialyltransferase‐III (ST3Gal‐III) caused by biallelic *ST3GAL3* gene variants are associated with human neurodevelopmental disorders. Although *ST3GAL3* gene variants have been linked to developmental and/or epileptic encephalopathy 15 (DEE15), their presence has only been reported in nine patients; however, the real frequency may be masked by insufficient screening.

**Methods:**

Phenotypic information was collected from a male patient with severe psychomotor developmental delay and epileptic seizures, and genetic testing was done using whole exome sequencing. A molecular dynamics simulation analysis was performed to assess the potential impacts of the identified *ST3GAL3* variants on the ST3Gal‐III protein function, and a literature review was conducted to compare this case with previously described cases and assess disease manifestation and genetic characteristics.

**Results:**

The patient inherited compound heterozygous *ST3GAL3* gene variants, NM_006279.5:c.809G>A (p.Arg270Gln) and c.921dupG (p.Thr308fs*8). Neither variant had been previously reported in the general population. The p.Arg270Gln variant disrupted a hydrogen bond in the simulated ST3Gal‐III protein structure. Among 25 patients with *ST3GAL3* gene defects, eight *ST3GAL3* gene variants were identified, and five variants had DEE signs.

**Conclusion:**

Patients with DEE15 may have novel *ST3GAL3* gene variants, and this study may be the first clinical report of their occurrence in a Chinese patient. These variants should be considered when evaluating patients presenting with unexplained early‐onset epileptic encephalopathy, severe developmental delay, and/or intellectual disability.

## Graphical abstract

Previous reports have found that ST3GAL3 variants are linked to developmental and/or epileptic encephalopathy (DEE); to date, it has only been reported in nine patients. We have reported on a patient who inherited compound heterozygous ST3GAL3 variants. The results will help to elucidate our understanding of the impacts of these variants in the future.

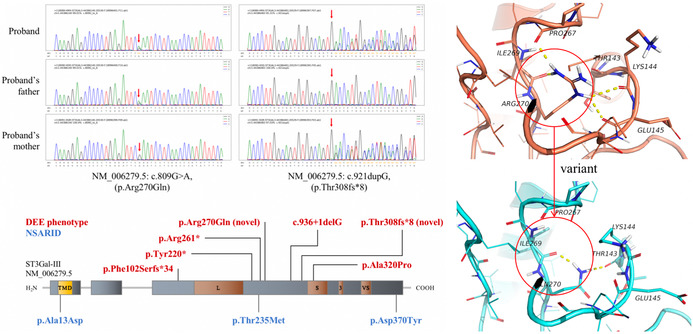

## 1 INTRODUCTION

Epileptic encephalopathy refers to a group of progressive psychomotor developmental disorders caused by abnormal discharges in the brain that severely affect cognitive behavior (Manole et al., 2023). Some patients may present with developmental disorders but do not have frequent epileptic seizures related to progressive motor developmental delay, and they are consequently classified as having developmental and/or epileptic encephalopathy (DEE). Patients with DEE typically have early onset, frequent seizures, and progressive motor developmental delay, characterized by phenotypes that cannot be reversed and may worsen, even if the seizures are controlled (Scheffer et al., 2017). DEE is predominantly caused by genetic factors, and the genes involved are primarily related to ion channels or proteins that control neuronal excitability. Furthermore, DEE shows highly heterogeneous characteristics, even in patients with the same variants or with different variants in the same genes (Guerrini et al., 2023).

The human *ST3GAL3* gene (NM_006279.2) encodes beta‐galactoside‐alpha‐2,3‐sialyltransferase‐III (ST3Gal‐III), a Golgi enzyme, which is highly expressed in the brain (Farajollahi et al., 2020). *ST3GAL3* was initially associated with nonsyndromic autosomal recessive intellectual disability, and later, *ST3GAL3* gene defects were identified in a family with West syndrome (Farajollahi et al., 2020; Hu et al., 2011). Currently, the *ST3GAL3* gene is associated with the intellectual developmental disorder, autosomal recessive 12 (MRT12, OMIM #611090), and DEE15 (OMIM #615006) in the OMIM database (https://omim.org/entry/606494?search=ST3GAL3&highlight=st3gal3). ST3Gal‐III is crucial for the normal brain development and function. Patients with DEE15 tend to have epileptic encephalopathy and epileptic features, whereas patients with MRT12 have severe intellectual disability (Farajollahi et al., 2020; Hu et al., 2011). However, to date, only nine cases of DEE associated with *ST3GAL3* gene variants have been reported, and more case reports are needed to enhance our understanding of the correlation between *ST3GAL3* gene variants and DEE phenotypes (Edvardson et al., 2013; Indellicato et al., 2020; Khamirani et al., 2021; Whitney et al., 2023).

Herein, we report a case of a male patient with *ST3GAL3* gene variants from a Chinese family, who presented with epileptic encephalopathy and psychomotor developmental delay. In addition, we have compared our findings in this case with those of previous research, summarizing the DEE phenotype and associated *ST3GAL3* genetic characteristics, to further highlight that *ST3GAL3* gene defects may be the causal agent in patients with epileptic encephalopathy.

## 2 MATERIALS AND METHODS

### 2.1 Ethics approval

The patient's parents signed an informed consent form, in which they agreed to disclose their child's clinical data and genetic information. This study was approved by the Medical Ethics Committee at the Hunan Children's Hospital (Changsha, China).

### 2.2 Genetic testing

Peripheral blood (3 mL) was separately collected from the proband and his parents for trio‐whole exome sequencing (WES). Genomic DNA was extracted from the samples using a Blood Genomic DNA Extraction Kit (CWBIO Biotechnology, Beijing, China), according to the manufacturer's instructions. After quality inspection, the DNA was fragmented using an ultrasonic treatment, and the xGEN® Exome Research Panel v1.0 probe (IDT, Coralville, IA, USA) was used to construct the exome sequencing library. The library was sequenced on a NovaSeq 6000 series high‐throughput sequencer (Illumina, San Diego, CA, USA). The raw data were subjected to end cleaning and then aligned to the human genome reference sequence v19 (GRCh37/hg19), using Burrows‐Wheeler Alignment software version 0.7.10. Variants were identified using Genome Analysis Toolkit software version 4.2.4.1, and ANNOVAR was used for annotation. Public variant databases, including dbSNP, 1000 Genomes Project, ExAC, and gnomAD, were queried to determine the frequency of variants in the general population. Online bioinformatics software programs, including SIFT, Polyphen‐2, and MutationTaster, were used to predict the biological impacts of the variants. The pathogenicity of the variants was evaluated according to the guidelines of the American College of Medical Genetics and Genomics (ACMG; Richards et al., 2015).

### 2.3 Sanger sequencing

After the amplification of the variants, Sanger sequencing was performed using an ABI 3500 analyzer (Applied Biosystems, MA, USA). The forward and reverse primer sequences for variant 1 were 5′‐FTCTCTTCACCATCATTCCCCTTG‐3′ and 5′‐CACTTCTGACCCAGCTTTCCTC‐3′, respectively. The forward and reverse primer sequences for variant 2 were 5′‐CTGAGATTCGAATCCTCAACCCA‐3′, and 5′‐ATGCGAACGGTCTCATAGTAGTG‐3′, respectively.

### 2.4 Molecular dynamics (MD) simulations

The protein structure was generated using the AlphaFold platform (https://alphafold.ebi.ac.uk/entry/Q11203), and the construction of the mutant structure was based on the wild‐type protein. MD simulations were performed using Gromacs 5.14 to generate trajectories and the Gromos 53a6 force field. The protein structure was placed in a cubic periodic box for the starting conformation, and the periodic boundary conditions of the simulation system were applied in the X/Y/Z directions with a minimum distance of 1.0 nm from the box boundary. Counterions were added to the solution to neutralize the protein charge. Energy minimization was performed using the steepest descent method for 400 steps, and each simulation system underwent a 50 ps position restraint simulation. The initial velocities for the formal MD simulations were randomly set. The results were visualized using PyMOL software version 2.5 and VMD, and the resulting figures were produced using Origin software version 8.5. The root mean square deviation (RMSD) of the protein was calculated using the gmx rms tool, and a scatter plot was drawn using Origin v8.5 software.

### 2.5 Literature review

The search period was from January 2010 to May 2023, and the studies were retrieved from PubMed (https://pubmed.ncbi.nlm.nih.gov/) and Europe PMC databases (https://europepmc.org/). The search key words included “ST3GAL3, Developmental Epileptic Encephalopathy 15, DEE15, beta‐galactoside alpha‐2,3‐sialyltransferase‐III.” Reports about patients with epileptic encephalopathy associated with *ST3GAL3* gene variants were collected. Studies without phenotype and genotype data as well as those about patients with isolated intellectual disabilities were excluded.

## 3 RESULTS

### 3.1 Phenotype presentation

**FIGURE 1:**
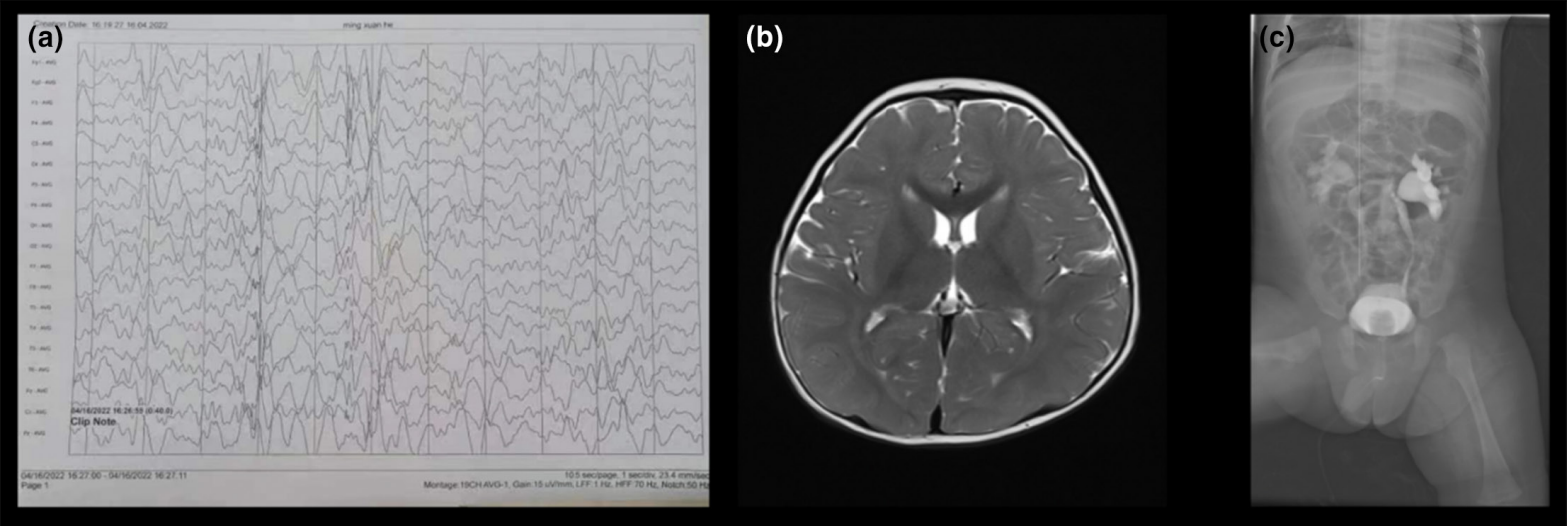
Clinical information of the patient. (a) Electroencephalogram showing an abundance of sharp low‐to‐high amplitude waves and 2.5–4 Hz spike‐and‐slow waves in the frontal and central regions during sleep. (b) Magnetic resonance image of the head showing no abnormalities. (c) Cystourethrography indicating the presence of the vesicoureteral reflux.

The patient was a 17‐month‐old male, the first child of his mother who had a history of one prior pregnancy. The patient weighed 5230 g at birth, with no history of asphyxia, and had Apgar scores of 10 at both 1 and 5 min. At 4 months of age, the patient exhibited difficulties in rolling over and following moving objects with his eyes. At 11 months of age, he was able to independently hold his head up and sit unassisted but was unstable. The patient had his first focal seizure at 6 months of age and has since experienced an average of one or two seizures per month. The seizure typically lasts for approximately 20 s. The main symptoms include clenched fists, jerking movements of the upper limbs, upward eye deviation, and unresponsiveness. The seizures were later controlled with sodium valproate. Video electroencephalogram monitoring revealed a slightly slowed background rhythm, frequent spikes, and sharp wave discharges in the frontal and central regions (Figure 1). The patient exhibited severe psychomotor retardation, characterized by the delayed development of both motor and cognitive functions. At 11 months of age, the patient's adaptability developmental age for adaptive behavior was 17 weeks, with a developmental quotient (DQ) of 35 (severe defect); for gross motor behavior was 15 weeks, with a DQ of 31 (extremely severe defect); for fine motor behavior was 21 weeks, with a DQ of 43 (moderate defect); for language behavior was 22 weeks, with a DQ of 45 (moderate defect); and for personal–social behavior was 15 weeks, with a DQ of 31 (severe defect). Upon physical examination during hospitalization, the patient exhibited poor functional movement, including an inability to turn over independently and limited ability to sit alone for only a few minutes. When assisted to a standing position, his legs needed brief support with hip flexion and inward foot rotation patterns and exhibited low muscle tone in the limbs. Magnetic resonance imaging of the head revealed no structural abnormalities. Bladder examination and ureterography revealed a vesicoureteral reflux (Figure 1).

The patient's parents were healthy, in a nonconsanguineous marriage, and reported no family history of inherited neurological developmental disorders, such as epilepsy or intellectual disability.

### 3.2 Genetic variant information

**FIGURE 2:**
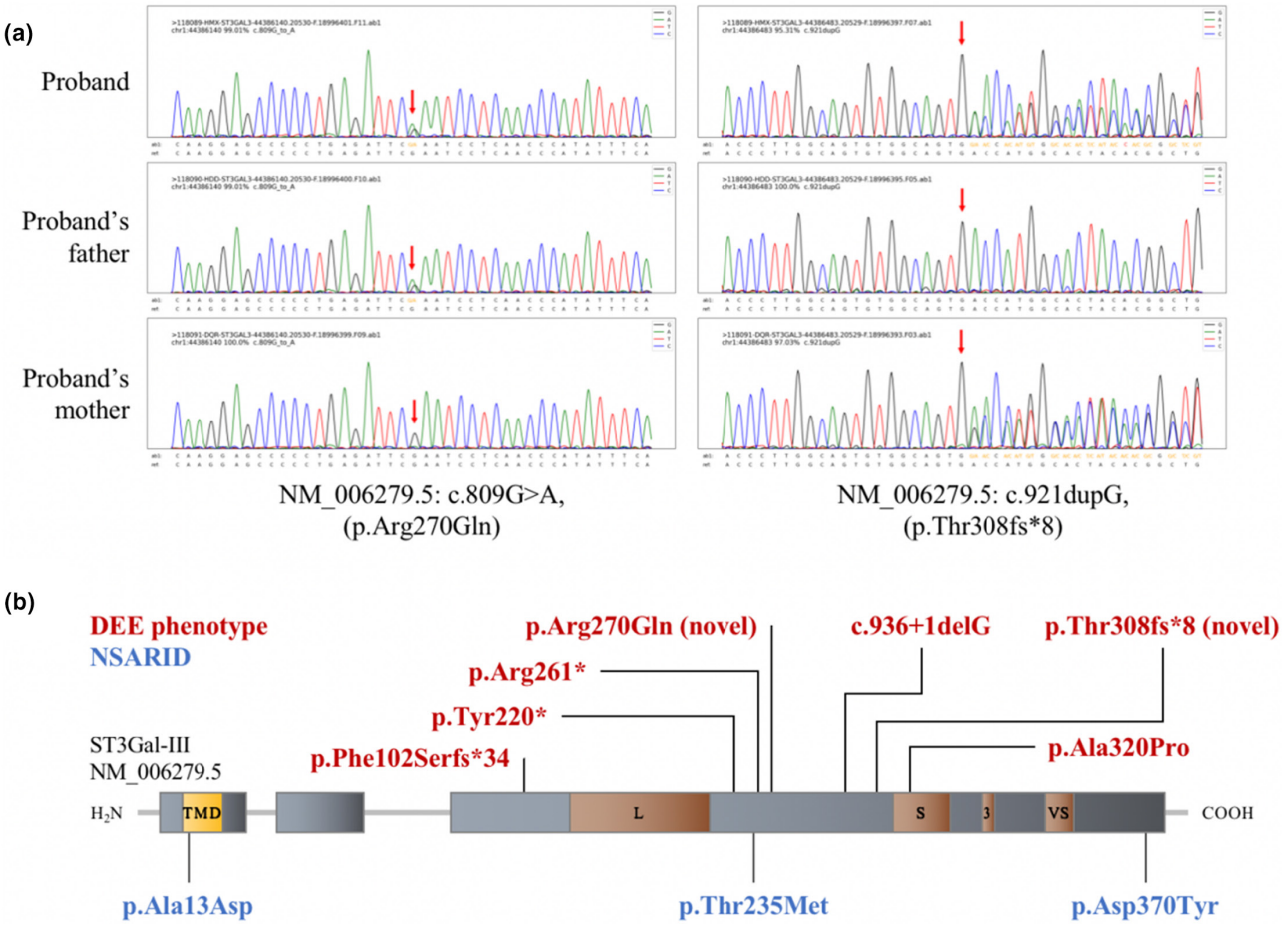
*ST3GAL3* gene variant information. (a) Sanger sequencing revealed compound heterozygous variants in the *ST3GAL3* gene of the patient inherited from his father and mother who carried heterozygous variants NM_006279.5:c.809G>A (p.Arg270Gln) and c.921dupG (p.Thr308fs*8), respectively. (b) Schematic of the ST3Gal‐III protein encoded by the *ST3GAL3* gene, including a short cytoplasmic domain near the NH_2_‐terminus, an amphipathic transmembrane domain (TMD), a stem region, and a catalytic domain near the COOH‐terminus, containing highly conserved motifs, such as large sialyl motif (L), short sialyl motif (S), motif 3, and very short sialyl motif (VS). To date, eight *ST3GAL3* gene variants have been reported (including two novel variants discovered in this study); those shown in red font are associated with developmental and epileptic encephalopathy (DEE) phenotypes, whereas those in blue font are associated with nonsyndromic autosomal recessive intellectual disability (NSARID) phenotypes.

WES generated a total of 55.2 million clean reads, with an average sequencing depth of 190 × and coverage of >20 × for 99.1% of the exome. Following the screening process, the patient was found to have compound heterozygous variants in the *ST3GAL3* gene, NM_006279.5:c.809G>A (p.Arg270Gln) and c.921dupG (p.Thr308fs*8), which were inherited from the patient's father and mother, respectively. These two variants were not found in dbSNP, 1000 Genomes, ExAC, or gnomAD, indicating that they were extremely rare in healthy populations. Several software programs, namely SIFT, Polyphen‐2, and MutationTaster, predicted that c.809G>A (p.Arg270Gln) would have a high probability of being damaging or deleterious. According to the ACMG criteria, the pathogenicity of p.Arg270Gln is of uncertain significance, with evidence that PM2_Supporting+PP3. p.Thr308fs*8 leads to a premature termination codon, and according to the ACMG criteria, it is likely pathogenic, with supporting evidence from PVS1 + PM2 (Table 1). Sanger sequencing confirmed the presence of these variants (Figure 2a).

**TABLE 1:** Summary data for the two *ST3GAL3* gene variants identified in this study.

No.	Nucleotide alteration	Amino acid change	1000 genomes	dbSNP	ExAC	gnomeAD	SIFT	Polyphen‐2_HDIV	Polyphen‐2_HVAR	MutationTaster	CADD	ACMG
1	c.809G>A	p.Arg270Gln	NA	NA	NA	NA	0.003 (Damaging)	1.0 (Probably_damaging)	0.987 (Probably_damaging)	0.956 (Disease_causing)	34 (Damaging)	Uncertain significance: PM2_Supporting+PP3
2	c.921dupG	p.Thr308fs*8	NA	NA	NA	NA	/	/	/	/	/	Likely pathogenic: PVS1 + PM2_Supporting

*Note*: /, no data.

Abbreviations: 1000Genome, https://www.internationalgenome.org/; ACMG, American College of Medical Genetics and Genomics; CADD, https://cadd.gs.washington.edu/; dbSNP, https://www.ncbi.nlm.nih.gov/snp/; ExAC/gnomeAD, https://gnomad.broadinstitute.org/; MutationTaster, https://www.mutationtaster.org/; NA, no account; Polyphen‐2, http://genetics.bwh.harvard.edu/pph2/; SIFT, https://sift.bii.a‐star.edu.sg/.

### 3.3 MD simulation results

**FIGURE 3:**
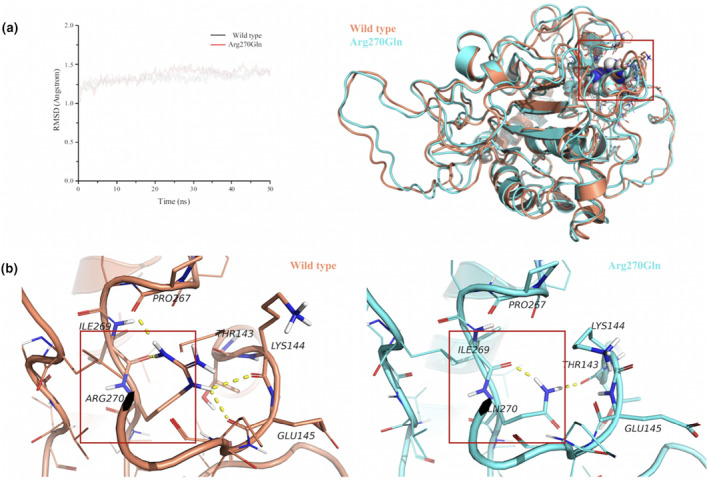
Structural analysis of the ST3Gal‐III variant protein. (a) Wild‐type (yellow) and mutant (blue) structures reach equilibrium at a root mean square deviation of approximately 1.5 Å. Red box in the right panel indicates the position of the variant Arg270Gln. (b) In the wild‐type structure, Arg270 with its positively charged guanidinium group forms stable hydrogen bonds with the surrounding residues Lys144, Glu145, Pro267, and Ile269. However, the variant p.Arg270Gln disrupts these hydrogen bonds, and therefore, Arg270 interacts only with Thr143 and Ile269, which may reduce the stability of the mutant protein (red box).

RMSD was calculated to assess the average deviation between conformations of the mutant and wild‐type proteins. Both the wild‐type and mutant structures reached equilibrium, with average RMSD values of approximately 1.5 Å. The interactions between the residues near Arg176 in the final binding state of the two systems were analyzed. In the wild‐type protein, Arg270 forms hydrogen bonds with Pro267, Ile269, Lys144, and Glu145. In the mutant structure, the original hydrogen bond interactions were disrupted, and new hydrogen bonds were formed between Ile269 and Thr143. This indicated that variant p.Arg270Gln might have altered the stability of the protein structure (Figure 3).

### 3.4 Literature review

Five articles have been found that contained data on a total of 25 patients, including information on their clinical manifestations and *ST3GAL3* variants (Edvardson et al., 2013; Farajollahi et al., 2020; Hu et al., 2011; Indellicato et al., 2020; Khamirani et al., 2021; Whitney et al., 2023). Among them, only nine patients (five males and four females) were reported to have DEE (Edvardson et al., 2013; Indellicato et al., 2020; Khamirani et al., 2021; Whitney et al., 2023). Most of the nine patients with DEE had their first seizure within 1 year of age (6/9), which progressed from West syndrome to Lennox–Gastaut syndrome (Edvardson et al., 2013). These patients showed signs of delayed psychomotor development before the onset of seizures, accompanied by varying degrees of language development delay and presence of severe intellectual disabilities. The efficacy of antiseizure treatments in these patients was generally low, with a majority experiencing uncontrolled seizures even with a combination therapy or increased dosages (Table 2).

**TABLE 2:** Clinical and phenotypic data of patients with DEE and *ST3GAL3* gene variants.

General			Variants			Age			Retardation			/					
Sub.	Sex	Consanguinity	Nucleotide	Amino acid	Zygosity	Diagnosis	Onset	Type	Psychomotor	Language	ID	EEG	MRI	AEDs	Control	Other	Ref.
1	M	+	c.958G>C	p.Ala320Pro	hom	2 y	3 m	Atonic	+	+	+	Hypsarrhythmia pattern	−	VGS, ACTH, VPA, CLB, LEV	Unsatisfactory	−	Edvardson et al., 2013
2	F	+	c.958G>C	p.Ala320Pro	hom	2 y	7 m	Atonic, tonic, myoclonic	+	+	+	Hypsarrhythmia pattern	−	VGS, ACTH, CLB, TPM, LEV, Rufinamide	VGS effective	−	
3	F	+	c.958G>C	p.Ala320Pro	hom	3 y	4 m	Generalized tonic–clonic	+	+	+	Hypsarrhythmia pattern	−	VGS, ACTH, CLB, CBZ, LEV, Rufinamide	VGS effective	−	
4	F	+	c.958G>C	p.Ala320Pro	hom	4 y	3 m	Focal clonic	+	+	+	Hypsarrhythmia pattern	−	VGS, ACTH, CLB, CBZ, LEV	VGS effective	−	
5	M	+	c.660C>A	p.Tyr220*	hom	18 m	7 m	Infantile spasms	+	+	+	Abnormal discharge of spike waves in the frontal lobe	T2 hyperintensity in the periaqueductal and posterior regions of the pons, mesencephalic region, and middle cerebellar peduncles, indicating the presence of restricted diffusion	VPA, ESM	Unsatisfactory	Plagiocephaly	Indellicato et al., 2020
6	F	+	c.660C>A	p.Tyr220*	hom	18 m	8 m	Infantile spasms	+	+	+	Epileptiform discharge	T2 hyperintensity in the periaqueductal and posterior regions of the pons, mesencephalic region, and middle cerebellar peduncles, indicating the presence of restricted diffusion	VPA, ESM	Unsatisfactory	Stereotyped behavior	
7	M	+	c.936 + 1delG	/	hom	12 y	3 y	Focal clonic	+	+	+	NA	−	VPA	Good	Autism, aggressive behavior, generalized hypotonia	Khamirani et al., 2021
8	M	−	c.302del	p.Phe102Serfs*34	het	12 y	5 y	Focal clonic	+	+	+	Focal sharp and spike‐and‐slow waves over the left temporal head region	T2 hyperintensity in the superior olivary nuclei, middle cerebellar peduncles, and dentate nuclei	CBZ	Good	−	Whitney et al., 2023
			c.781C>T	p.Arg261*													
9	M	−	c.302del	p.Phe102Serfs*34	het	10 y	2.5 y	Focal clonic	+	−	−	Single tiny focus of deep white matter signal abnormality in the left frontoparietal subcortical region	−	CBZ + LEV	Good	−	
			c.781C>T	p.Arg261*													
Our patient	M	−	c.809G>A	p.Arg270Gln	het	17 m	6 m	Focal clonic	+	+	+	Frequent occurrence of spike waves and sharp slow waves in the frontal lobe and central region	−	VPA	Good	Generalized hypotonia	
			c.921dupG	p.Thr308fs*8													

*Note*: +, phenotype; −, no phenotype; /, no data.

Abbreviations: ACTH, adrenocorticotropic hormone; AEDs, antiepileptic drugs; CBZ, carbamazepine; CLB, clobazam; EEG, electroencephalogram; ESM, ethosuximide; F, female; het, heterozygote; hom, homozygote; ID, intellectual disability; LEV, levetiracetam; M, male; m, month; MRI, magnetic resonance imaging; NA, no account; TPM, topiramate; VGB, vigabatrin; VPA, valproate; y, year.

Eight *ST3GAL3* variants have been reported, and they are primarily concentrated in the glycosyltransferase family 29 domain of ST3Gal‐III (Schnaar et al., 2014). The DEE‐related variants included c.660C>A (p.Tyr220*), c.936 + 1delG, c.958G>C (p.Ala320Pro), c.302del (p.Phe102Serfs*34), and c.781C>T (p.Arg261*) (Edvardson et al., 2013; Indellicato et al., 2020; Khamirani et al., 2021; Whitney et al., 2023; Figure 2b).

## 4 DISCUSSION

To the best of our knowledge, we possibly presented the first documented case of DEE15 within the Chinese population and identified novel *ST3GAL3* gene variants, which may serve as pathogenic factors contributing to severe psychomotor developmental delay, delayed motor milestones, seizures, and intellectual disability. The phenotype of the patient was consistent with the characteristics described in previous clinical studies. Patients with DEE and *ST3GAL3* gene variants are very rare, and to date, only nine such patients have been identified. These patients exhibited severe psychomotor developmental delay and had delayed motor development milestones in early life. Although some patients showed slight improvements over time, they did not achieve normal developmental levels. Additionally, most patients have intractable epilepsy, language developmental delays, autism, or aggressive behavior (Edvardson et al., 2013; Indellicato et al., 2020; Khamirani et al., 2021). The majority of patients, including the patient described in this report, experienced their first seizure before the age of one, primarily presenting as a focal seizure. However, it appears that the seizure type and drug control are not related to genotype, and even within the same family, the sensitivity to antiepileptic drugs may vary among individuals. Some patients may experience improvement after treatment with vigabatrin or sodium valproate. However, even if seizures are controlled, these patients still experience severe psychomotor developmental delays and intellectual disability. Therefore, the treatment of patients requires a comprehensive approach that not only controls epilepsy but also involves targeted rehabilitation training.

The *ST3GAL3* gene is located on chr1p34.1 and consists of 15 exons. ST3Gal‐III is a sialyltransferase located in the Golgi apparatus. Structurally, the N‐terminal cytoplasmic tail of ST3Gal‐III is connected to an amphipathic transmembrane domain structure, which plays an important role in accurately positioning the Golgi apparatus. The C‐terminal catalytic region is composed of multiple highly conserved sialyl motifs (Hu et al., 2011). ST3Gal‐III mediates the transfer of sialic acid residues to oligosaccharide chains of various glycoproteins and glycolipids and is an important regulator of cell functions and information transmission (Wang et al., 2017). ST3Gal‐III dysfunction affects multiple physiological parameters, such as neuronal migration, synaptic plasticity, and neuronal apoptosis, indicating that the *ST3GAL3* gene plays a critical role in human neural development (Hu et al., 2011; Wang et al., 2017).

In addition to their association with DEE15, *ST3GAL3* defects can lead to MRT12 (OMIM #611090), which is characterized by nonsyndromic severe intellectual disability without seizures or early psychomotor developmental delays (Farajollahi et al., 2020; Hu et al., 2011). However, the mechanisms through which *ST3GAL3* defects cause these conditions remain unclear. The residual level of ST3Gal‐III enzymatic activity may play a role in pathological manifestations. It is known that accurate positioning in the Golgi apparatus and binding to its substrate are prerequisites for ST3Gal‐III enzyme activation, and the incorrect subcellular localization may affect ST3Gal‐III synthesis and activation process (Eckhardt et al., 1996; Tu & Banfield, 2010). Hu et al. (2011) reported that p.Ala13Asp mutant proteins were mislocalized to the endoplasmic reticulum, resulting in a slight decrease in enzyme activity. ST3Gal‐III harboring another variant, p.Asp370Tyr, was completely mislocalized to the endoplasmic reticulum but retained a small amount of enzyme activity. Both variants were associated with severe intellectual disability in patients without seizure symptoms (Hu et al., 2011). ST3Gal‐III with the variant p.Ala320Pro, reported by Edvardson et al. (2013) was completely mislocalized and had no enzymatic activity, leading to a severe case of West syndrome. Van Diepen et al. (2018) suggested that p.Ala320Pro caused a complete loss of enzyme activity owing to the restriction of the proline dihedral angle at the phi position, which significantly interfered with protein structural stability. Notably, the variant p.Tyr220* reported by Indellicato et al. (2020) also caused severe seizure symptoms; however, ST3Gal‐III with this variant was not mislocalized to the endoplasmic reticulum, like proteins bearing other variants, but accumulated in the Golgi apparatus, leading to rapid degradation of truncated protein. These functional studies of variants indicate that *ST3GAL3* gene‐related seizure symptoms are primarily related to disturbances in enzymatic activity rather than incorrect subcellular localization. Therefore, the loss‐of‐function variants are more likely to cause DEE. Notably, two of the three *ST3GAL3* gene variants reported to be associated with seizures are loss‐of‐function variants, p.Tyr220* and c.936 + 1delG (Indellicato et al., 2020; Khamirani et al., 2021).

In previous reports, the majority of patients had homozygous variants in the *ST3GAL3* gene, as a result of consanguineous marriages. However, in this study, compound heterozygous variants of the *ST3GAL3* gene, NM_006279.5: p.Thr308fs*8 and p.Arg270Gln, were identified. Neither variant had been previously reported in the normal population. The former is a frameshift variant that causes premature termination of codons, and the NMDetective data model developed by Lindeboom et al. (2019) predicted that this variant may trigger nonsense‐mediated mRNA decay (NMDetective score = 0.60; Supek et al., 2021). The latter is a missense variant located in the C‐terminal catalytic loop. The substitution of positively charged arginine with uncharged glutamine may alter interactions with surrounding residues and increase local structural hydrophobicity. MD simulations predicted that this variant would disrupt the hydrogen bonds between the mutated residue and surrounding residues, leading to changes in local structural stability. Notably, although the patient in this study exhibited severe psychomotor developmental delay, seizures were controlled with sodium valproate. Further investigation is required to determine whether the mild seizure phenotype was owing to the residual enzyme activity of the protein with p.Thr308fs*8 and p.Arg270Gln variants.

In summary, we reported a rare case of DEE with severe psychomotor developmental delays and seizures and identified two novel variants of the *ST3GAL3* gene that are associated with this condition. This study highlights the importance of genetic testing and counseling for patients with epilepsy and epileptic encephalopathy.

## AUTHOR CONTRIBUTIONS

Juan Liu wrote the draft. Jihong Hu, Juan Liu, and Chunguang Guo performed the genetic analysis. Jihong Hu edited the final article. Chunguang Guo, Yaqin Duan, and Chunlei Liu collected the clinical information. Yaqiong Tan and Ying Pan performed the data analyses. All authors agreed to be accountable for and ensure any questions relating to the accuracy and integrity of this work and read and approved the final article.

## FUNDING INFORMATION

This research was supported by Hunan Provincial Department of Science and Technology—Clinical Medical Research Center Project (ID: 2021SK4018), Hunan Provincial Federation of Disabled Persons Project (ID: 2019XK002).

## CONFLICT OF INTEREST STATEMENT

The authors declare that the research was conducted in the absence of any commercial or financial relationships that could be construed as a potential conflict of interest.

## ETHICS STATEMENT

The study was approved by the ethics committee of the Hunan Children's Hospital.

## CONSENT

Written informed consent was provided by the participant.

## Data Availability

The data that support the findings of this study are available from the corresponding author upon reasonable request.
